# The Role of Interventional Pain Management in Proteus Syndrome: A Case Report

**DOI:** 10.7759/cureus.24651

**Published:** 2022-05-01

**Authors:** Ben Silverman, Gurtej Bajaj, Eric Liu, Adison Weseloh, Adrian Popescu

**Affiliations:** 1 Physical Medicine and Rehabilitation, University of Pennsylvania, Philadelphia, USA

**Keywords:** overgrowth syndrome, flouroscopic injection, scoliosis, zygapophyseal joint, proteus syndrome

## Abstract

Proteus syndrome (PS) is a rare overgrowth disease process with only a few hundred cases being reported in the literature. Abnormal formation of the vertebral bodies causing scoliosis and spinal stenosis are common features that lead to debilitating pain in these patients. We present a case of a 35-year-old male landscaper with a history of PS causing severe scoliosis and vertebral overgrowth who underwent recurrent sets of multilevel zygapophyseal joint injections for management of his axial back pain. This case illustrates the utility of interventional spinal procedures in patients with progressive pain from PS.

## Introduction

Proteus syndrome (PS) is a rare condition that is characterized by excessive growth of various parts of the human body. It was first described in 1979 and later termed PS in 1983 after the Greek sea-god Proteus, who had the ability to change shapes [1]. This syndrome is seen in less than one in one million people and is caused by mutations in the AKT1 gene [2]. The AKT1 gene ultimately codes for a protein that helps regulate cell growth and division. A mutation in this gene can therefore lead to increased rates of cell growth as well as prevention of cell death [3]. This mutation occurs spontaneously without a notable inheritance pattern but is seen to follow a mosaic process. 90% of people with PS are found to have this genetic mutation [4]. This syndrome can thereby affect people differently and lead to a wide array of phenotypes including osseous overgrowth of the skin, adipose tissue, skeletal muscle, and the central nervous system [5]. Its manifestations are seen from toddlerhood onward causing progressive disfigurement, a wide variety of tumors, and most commonly pain. The overgrowth in PS does not typically manifest until around six to 18 months, with some cases reporting onsets as late as 12 years old [6]. Specifically, about the spinal vertebral bodies, its asymmetric overgrowth at various levels predisposes patients to potentially debilitating pain that could warrant several procedural or surgical interventions.

## Case presentation

**Figure 1:**
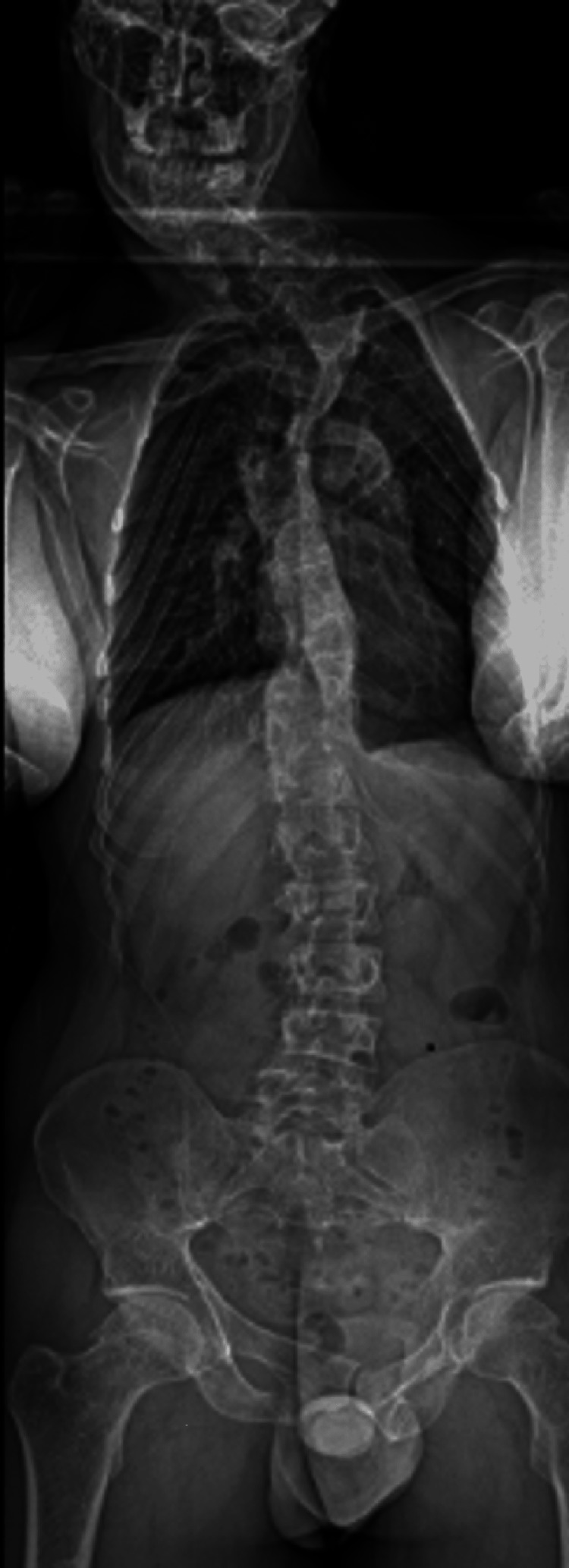
Anterior posterior (AP) view of erect thoracolumbar scoliosis series with severe levoscoliosis centered at the cervicothoracic junction, moderate dextroscoliosis of the upper thoracic spine and mild levoscoliosis of the lower thoracic spine.

**Figure 2:**
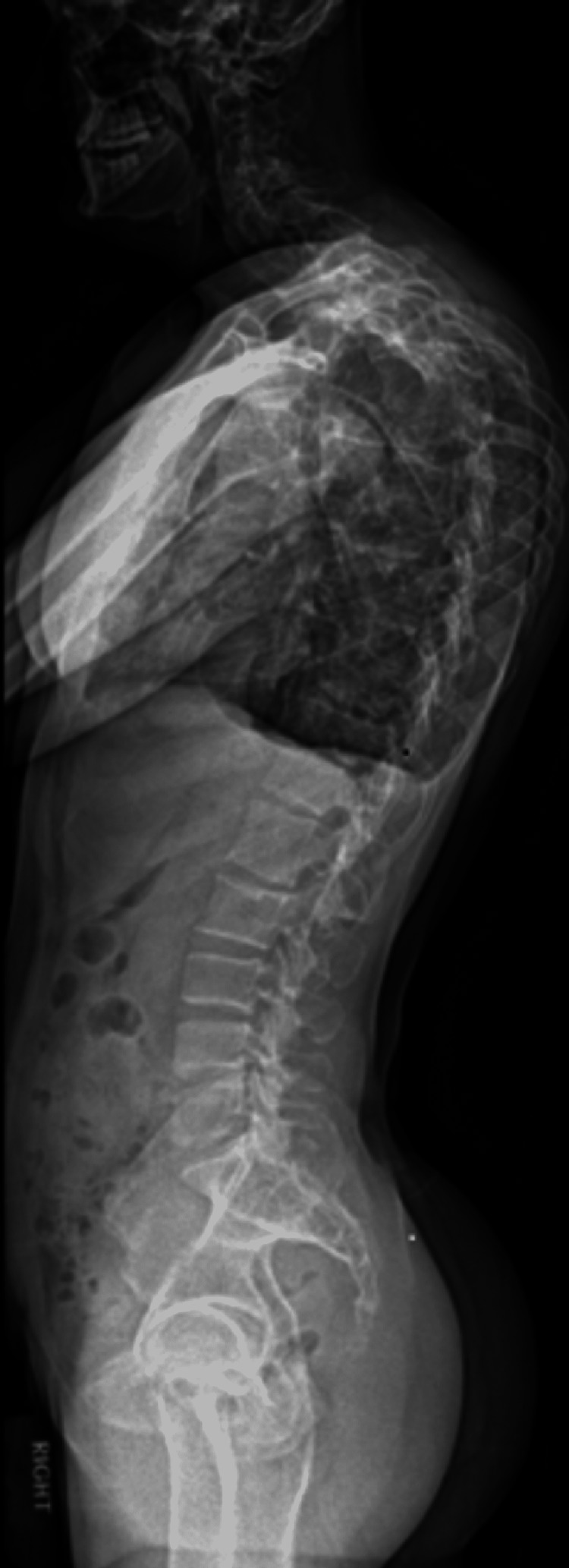
Lateral view of erect thoracolumbar scoliosis series with severe levoscoliosis centered at the cervicothoracic junction, moderate dextroscoliosis of the upper thoracic spine and mild levoscoliosis of the lower thoracic spine.

A 35-year-old hardworking male with a career in landscaping presented to an outpatient spine center in 2019 with back pain. Past medical history revealed he was diagnosed with PS at the age of 10 months. The diagnosis was made following episodes of seizures and upon workup was found to have numerous benign tumors including hamartomas, osteomas, and lipomas. His pain at this visit was described as left-sided thoracic spinal pain that had been gradually worsening over a few months. The pain was achy with no specific exacerbating factors. The physical exam was notable for paraspinal tenderness under the apex of the left scapula. No signs of myelopathy were revealed at that time. The patient had been previously prescribed medications including non-steroidal anti-inflammatories, muscle relaxants, and opioids with no relief. Initial imaging with erect thoracolumbar scoliosis of the cervicothoracic junction series demonstrated severe levoscoliosis, defined as a curvature of the spine to the left. Moderate dextroscoliosis, defined as a curvature of the spine to the right, and mild levoscoliosis were demonstrated in the upper thoracic spine and lower thoracic spine, respectively (Figures 1, 2).

**Figure 3:**
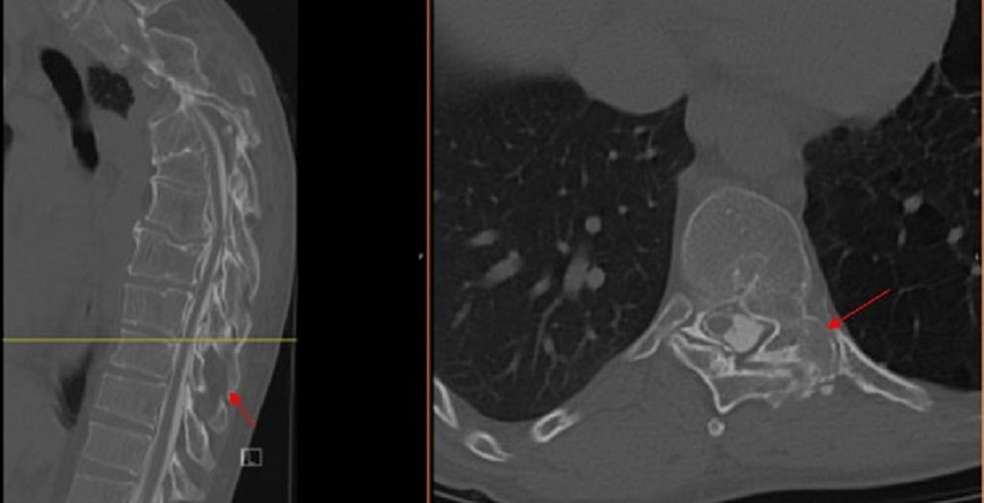
T8-9 sagittal (left) and axial (right) views. Arrows depicting moderate spinal canal stenosis, due to posterior disc osteophyte complex and ligamentum flavum/thickening/ossification.

**Figure 4:**
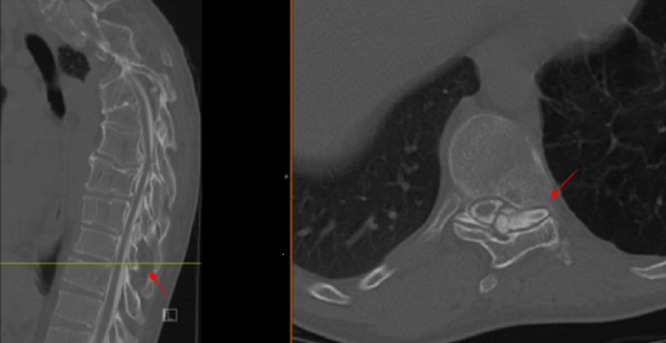
T9-10 sagittal (left) and axial (right) views. Moderate spinal canal stenosis, secondary to marked left facet arthropathy.

Due to his history of PS, a CT myelogram was performed which demonstrated multilevel osseous overgrowths in the spine with multilevel areas of osseous fusion most pronounced in the cervicothoracic spine (Figures 3, 4).

**Figure 5:**
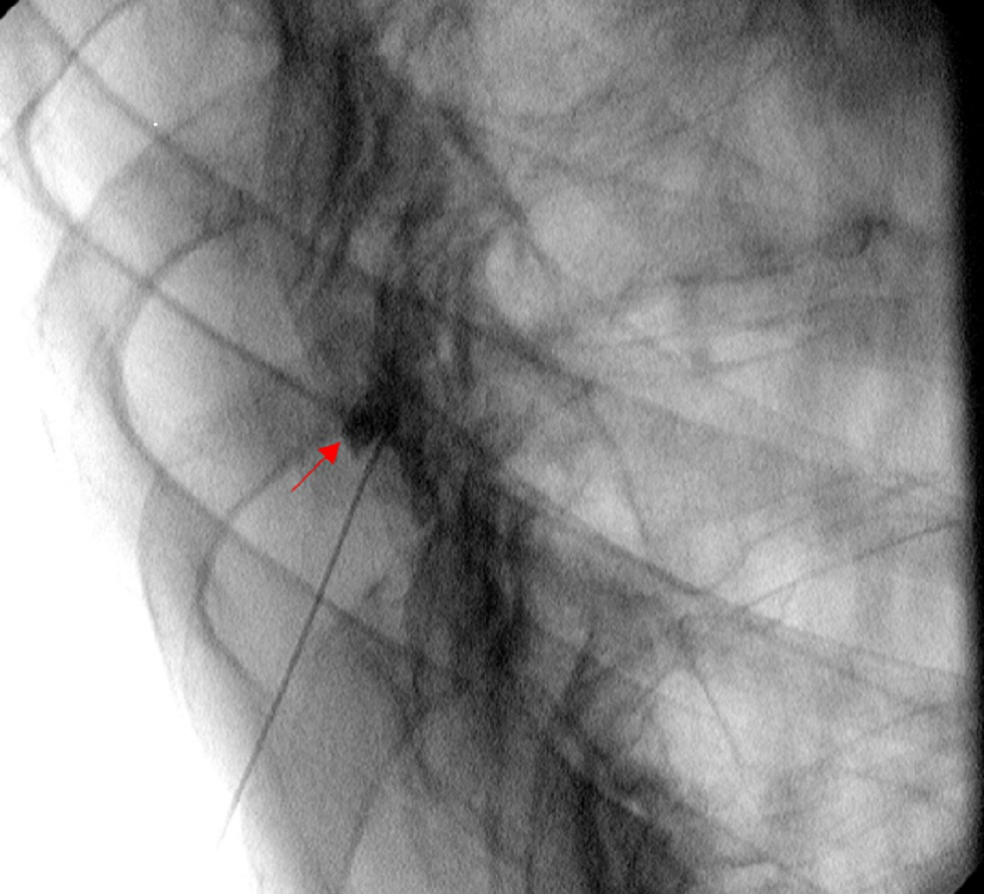
Oblique view: Fluoroscopic guided left T9-T10 zygapophysial joint steroid injection.

**Figure 6:**
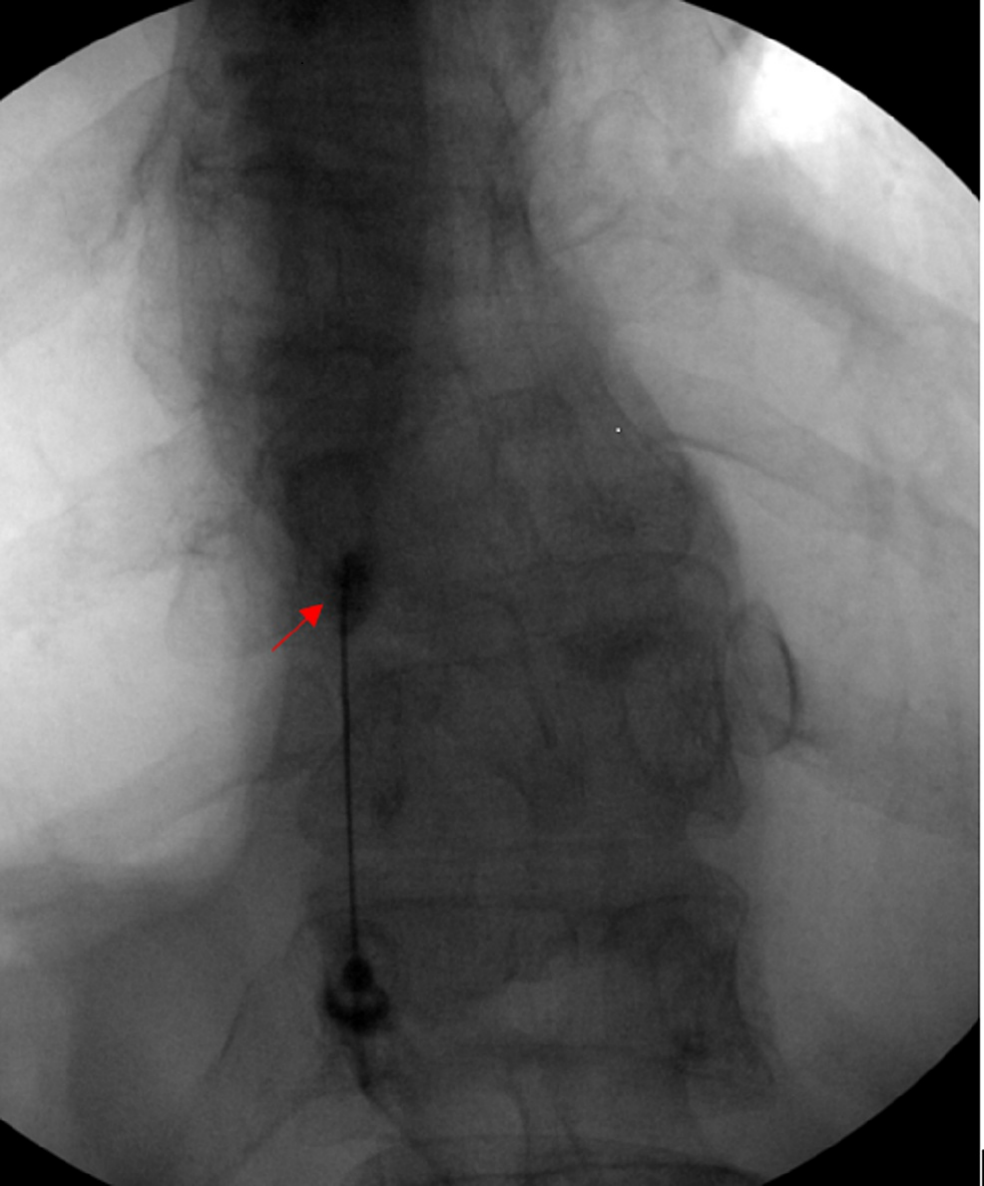
AP view: Fluoroscopic guided left T9-T10 zygapophysial joint steroid injection.

The patient opted for a non-surgical approach as he was not interested in a spinal fusion at this time. He underwent left T8-T9 and T9-T10 facet joint injections as well as a T8-T9 costotransverse injection (Figures 5, 6).

These injections contained 20 mg of dexamethasone and 1.5 mL of 1% lidocaine for inflammation and pain. Correct placement of the target location was obtained by using 0.5 mL of iopamidol as contrast. In addition, the patient participated in a course of physical therapy focused on strengthening and stretching of his abdominal and back musculature. Using the numerical rating scale, his pain was noted to be an 8 prior to the procedures, with a post-procedure pain score of two, which lasted up to six months later. Given the decrease in pain and improvement in quality of life over this period, he underwent repeat spinal procedures two weeks later. He had been doing well until about one year later when he presented with increased back pain that was now limiting his activities of daily living and requiring him to stop his landscaping career. He further underwent a third set of repeat injections one week later with additional discussions for an extensive fusion of his spine if pain was to progress.

## Discussion

As previously described, PS is a rare overgrowth disease process with a wide range of malformations, many of which can lead to axial back pain. This syndrome can be associated with physical deformities that can not only lead to crippling pain but also reduced quality of life. Decreased independence and functional status, specifically the ability to work, appeared to be one of the main factors that led this patient to seek treatment.

Despite limited literature, the chronic pain and decrease in quality of life appear to stem from PS causing overgrowth of the vertebral bodies leading to severe scoliosis and spinal stenosis [7]. Compromise of the spinal cord in patients with PS can result from canal narrowing secondary to vertebral hypertrophy or infiltration of the spinal canal by an angiolipomatous mass [8]. Surgical interventions for those with noted kyphosis and vertebral overgrowth have even been described in the literature with improved functionality and decreased pain [9]. Despite reports of positive surgical outcomes, there appears to be a noted paradox in treating patients surgically. Surgical intervention is thought to be delayed until it is deemed necessary due to the severe complications that can occur in this syndrome, including pulmonary embolism and deep venous thrombosis [10]. Given severe complications from surgery, physicians have looked to more conservative forms of treatment including physical therapy and non-operative spinal procedures. This case highlights the use of repetitive zygapophysial injections as the possible management of recurrent and progressive axial pain related to PS. However, even with months of pain relief following these injections, the pain in PS can be recurrent, which can make treatment challenging. As some patients look to avoid surgery, facet injections may be a good consideration for the initial management of axial pain caused by this syndrome. Conservative management can be without the severe complications related to PS; however, if pain continues to progress, surgical consultation may be eventually needed. Additional cases should be further studied to provide greater awareness of this potentially debilitating syndrome.

## Conclusions

This case report highlights the use of interventional spinal procedures, specifically zygapophyseal joint injections to manage axial back pain related to PS. This patient had debilitating back pain that was hindering his quality of life. These spinal injections provided repeated pain relief for up to six months at a time which allowed increased mobility and functional status. Continued follow-up will be needed to assess if these injections can continue to decrease his pain and also improve his quality of life over a longer period of time.
